# CHROMATIN STATE AND MODIFICATIONS IN AGING

**DOI:** 10.1093/geroni/igac059.102

**Published:** 2022-12-20

**Authors:** Simone Sidoli

## Abstract

Program Overview: In this symposium, the speakers will discuss how genomic stability and chromatin state are affected during aging using complementary perspectives, model systems and technology. Methods such as structural genomics, genome-wide mapping, super-resolution microscopy, mass spectrometry, and enzymology are utilized to describe hallmarks of aging in models such as C. elegans, mice, and 2D vs 3D human cell culture. Dr. Lakadamyali applies innovative super-resolution microscopy to visualize and quantify the spatial organization of chromatin with nanoscale spatial resolution in single cells, revealing how disordered patches at the nucleosomal level correlate with cell phenotype. Dr. Sen will present a multi-omics approach on murine models to reveal that aged cells organize a specific type of heterochromatin unable to self-interact over long distances. These findings expose that global chromatin alterations pose a barrier and promote a cellular state that is refractory to injury sensing and repair. Finally, Dr. Sidoli will describe how mass spectrometry is applied to investigate combinatorial histone modifications that benchmark regions of decondensed heterochromatin, and show the enrichment of unusual histone modifications in a cohort of centenarian patients.

